# Audiometric thresholds and auditory processing in children with early malnutrition: a retrospective cohort study

**DOI:** 10.1590/1516-3180.2014.1325686

**Published:** 2014-07-22

**Authors:** Patricia Aparecida Zuanetti, Maria Fernanda Laus, Adriana Ribeiro Tavares Anastasio, Sebastião de Sousa Almeida, Marisa Tomoe Hebihara Fukuda

**Affiliations:** I BSc. Doctoral Student of Audiology, Department of Ophthalmology, Otorhinolaryngology and Head and Neck, Faculdade de Medicina de Ribeirão Preto (FMRP), Universidade de São Paulo (USP), Ribeirão Preto, Brazil; II BSc. Doctoral Student and Nutritionist, Department of Psychology, Faculdade de Filosofia Ciências e Letras de Ribeirão Preto (FFCL), Universidade de São Paulo (USP), Ribeirão Preto, Brazil; III BSc, PhD. Associate Professor, Department of Ophthalmology, Otorhinolaryngology and Head and Neck, Faculdade de Medicina de Ribeirão Preto (FMRP), Universidade de São Paulo (USP), Ribeirão Preto, Brazil; IV BSc, PhD. Associate Professor, Department of Psychology, Faculdade de Filosofia Ciências e Letras de Ribeirão Preto (FFCL), Universidade de São Paulo (USP), Ribeirão Preto, Brazil

**Keywords:** Malnutrition, Child nutrition disorders, Hearing, Ear, inner, Hearing disorders, Desnutrição, Transtornos da nutrição infantil, Audição, Orelha interna, Transtornos da audição

## Abstract

**CONTEXT AND OBJECTIVE:**

Malnutrition is one of the causes of changes in cell metabolism. The inner ear has few energy reserves and high metabolism. The aim of this study was to analyze whether malnutrition at an early age is related to impairment of auditory processing abilities and hearing abnormalities.

**DESIGN AND SETTING:**

Retrospective cohort study conducted in a tertiary public hospital.

**METHODS:**

45 children participated, divided as follows: G1, children diagnosed with malnutrition in their first two years of life; G2, children without history of malnutrition but with learning difficulties; G3, children without history of malnutrition and without learning difficulties. Tympanometry, pure-tone audiometry and the Staggered Spondaic Word (SSW) test (auditory processing) were performed. Statistical inferences were made using the Kruskal-Wallis test (α = 5%) and the test of equality of proportions between two samples (α = 1.7%).

**RESULTS:**

None of the 45 children participating in this study presented hearing deficiencies. However, at six of the eight frequencies analyzed, the children in G1 presented hearing thresholds lower than those of the other groups. In the auditory processing evaluation test, it was observed that 100% of the children in G1 presented abnormal auditory processing and that G1 and G2 had similar proportions of abnormalities (P-values: G1/G2 = 0.1; G1/G3 > 0.001; G2/G3 = 0.008).

**CONCLUSIONS:**

Malnutrition at an early age caused lowering of the hearing levels, although this impairment could not be considered to be a hearing deficiency. Every child in this group presented abnormalities in auditory processing abilities.

## Abstract (pt)

**CONTEXTO E OBJETIVO:**

A subnutrição é uma das causas das alterações no metabolismo celular. A orelha interna possui poucas reservas energéticas e alto metabolismo. O objetivo deste estudo foi de analisar se a subnutrição em idade precoce está relacionada a prejuízos em habilidades do processamento auditivo e a alterações auditivas.

**TIPO DE ESTUDO E LOCAL:**

Estudo de coorte retrospectivo conduzido em hospital público terciário.

**MÉTODOS:**

Participaram 45 crianças divididas em: G1, crianças que tiveram o diagnóstico de subnutrição nos dois primeiros anos de vida; G2, crianças sem histórico de subnutrição, mas com dificuldades de aprendizagem; G3, crianças sem histórico de subnutrição e sem dificuldades escolares. Realizou-se a timpanometria, audiometria tonal limiar e o teste de SSW [Staggered Spondaic Word] (processamento auditivo). Para a inferência estatística, usou-se o Kruskal-Wallis (α = 5%) e o teste igualdade de proporções entre duas amostras (α = 1,7%).

**RESULTADOS:**

Nenhuma das 45 crianças participantes neste estudo apresentou deficiência auditiva, porém em seis das oito frequências avaliadas, as crianças do G1 apresentaram limiares auditivos rebaixados quando comparadas às de outros grupos. No teste que avaliou o processamento auditivo, constatou-se que 100% das crianças do G1 possuem alteração do processamento auditivo, e que G1 e G2 possuem proporção semelhante de presença de alterações (valor de P: G1/G2 = 0,1; G1/G3 = > 0,001; G2/G3 = 0,008).

**CONCLUSÕES:**

A subnutrição em idade precoce causou rebaixamento nos níveis de audição, apesar de este prejuízo auditivo não ser considerado como deficiência auditiva. Todas as crianças deste grupo possuem alterações em habilidades do processamento auditivo.

## INTRODUCTION

The first years of life are considered to be the most important ones for the development of hearing and language skills.^1^ Rapid brain growth occurs from the second trimester of gestation to the second year of life,^2^ together with accelerated neurogenesis, gliogenesis, neuronal migration and myelinization processes.^1^ ^,^ ^3^ Thus, in order to prevent impairment of the auditory pathways or of other skills relating to language during this period, the child must be exposed to appropriate stimulation. Moreover, no type of intercurrence should occur, since these events may often lead to irreversible cognitive damage at a later time.^3^

In this respect, malnutrition may play an important role, since although its relationship with hearing changes has been little explored at either the peripheral or the central level, it may interfere with the metabolism of the inner ear, thus causing hearing loss.^4^ Malnutrition is characterized as a lack of nutrients that are necessary for the physiological metabolism of the organism (chemical reactions that occur in the cells of the body), due to energy expenditure that is greater than the ingested energy.^3^ The damage caused by malnutrition to the growth and development of the organism depends on the type of malnutrition, the duration of this disease, its severity and other aggravating factors such as infections and genetic factors, in addition to the individual's age at the time of onset of this condition.^5^

Studies investigating the interference of malnutrition with the development of the auditory pathways have used brainstem auditory evoked potential (BAEP) as an evaluation instrument. Such studies have observed that malnutrition causes delayed maturation of the auditory pathways, as represented by elevated absolute and interpeak latencies.^6^ ^,^ ^7^ The set of auditory skills on which individuals depend in order to interpret what they hear (skills such as sound localization, auditory discrimination, recognition of time patterns, temporal resolution, temporal integration and organization) is defined as auditory processing and involves central structures ranging from the cochlear nuclei to areas of the cortex.^8^ Children with a history of middle ear abnormalities or some type of hearing deficiency, and those who have been diagnosed as presenting abnormalities of auditory processing are at risk of presenting difficulties with oral and written language. 

There is evidence that malnutrition has an effect on peripheral and central hearing and on the development of auditory pathways, Furthermore, integrity of the auditory system is important for the development of oral and written language. However, in both the Brazilian and the worldwide literature, there are only a few studies on how malnutrition affects the peripheral and central auditory system. Nonetheless, this subject is very important in relation to the development of children's language, and detailed study on the relationship between malnutrition and the auditory system is needed, using other evaluation methods.

## OBJECTIVE

To analyze whether malnutrition at an early age is related to damage to auditory processing skills and to changes in hearing thresholds.

## METHODS

### Ethical issues

The study was approved by our institution's Research Ethics Committee, under protocol number 1924/2009.

### Study design and sample selection

This was a retrospective cohort study carried out at a tertiarylevel public hospital. The participants were 45 children (21 girls and 24 boys) aged 7 to 10 years (mean ± SD: 8.2 ± 0.7) divided into three groups: G1 (n = 15), consisting of children with a history of malnutrition at an early age; G2 (n = 15), consisting of children without any history of malnutrition and with learning difficulties; and G3 (n = 15) consisting of children with no history of malnutrition and no learning difficulties. The children in the three groups were matched according to the variables of age and type of school institution in the proportions of 1:1:1, in which G1 children were considered to be the reference category.

The exclusion criteria for all three groups were as follows: not attending school regularly; presence of syndromes that impaired cognitive function; no type A tympanometric curve on the day of the audiological examination; conductive or mixed hearing loss, taking into consideration frequencies from 500 to 4000 Hz; and presence of primary neurological abnormalities due to malnutrition for G1 or a history of malnutrition or altered nutritional status on the date of the evaluation for G2 and G3.

The inclusion criteria for G1 were as follows: a diagnosis of moderate or severe malnutrition during the first two years of life (diagnosed by the medical team of the hospital and described in the subjects' medical records); and a history of nutritional rehabilitation after malnutrition, regardless of current nutritional status.

### Sample selection and characterization

To select the G1 subjects, we analyzed 548 medical records at Hospital das Clínicas (HC), Faculdade de Medicina de Ribeirão Preto (FMRP), of children who had spent some time hospitalized during their two first years of life with a diagnosis of malnutrition. Only 79 children fulfilled the inclusion and exclusion criteria of the study and only 15 came for evaluation. The following data in the medical records were analyzed: date of the medical diagnosis of malnutrition; anthropometric data obtained at the time of diagnosis; child's age at the time of diagnosis; degree of malnutrition at the time of diagnosis; and time elapsed between the diagnosis of malnutrition and the diagnosis of eutrophy or of no remaining evidence of malnutrition.

The School Performance Test^9^ was used to assess the school performance of students regularly enrolled in the public elementary school network, in order to form G2 and G3. These students were selected at random for evaluation in the reading, writing and arithmetic tests of the SPT. 

The three groups underwent anamnesis consisting of questions about the children's development and medical history. Their nutritional status was also evaluated, for which the following anthropometric measurements were used: Weight (W): body weight, in kg, was measured by means of a Bal-Isopa TecLine digital scale with 0.1 kg resolution. This procedure was performed in the morning under fasting conditions, with each subject wearing light clothing, barefoot and having voided his/her bladder. Height (H): the subjects stood up in an erect position with their head in the vertical plane, barefoot and with their feet close together, and supporting their back, buttocks and heels against the wall, on which a mark was made and the value in cm was measured with a metric tape with 1 cm graduations. 

The Z-score for weight/age (W/A) was used for diagnosing the previous nutritional status of G1 children and the Z-score for body mass index (BMI)/age (BMI/A) was used for the current anthropometric evaluation of all children (G1 and G2). A Z-score of less than -2 was taken to demonstrate malnutrition. A professional nutritionist calculated and analyzed the indicators using the Epi Info software. 

### Peripheral and central auditory assessment 

All the children underwent audiological assessment, which consisted of the procedures listed below.

### Tonal threshold audiometry

The Midimate 622 instrument (Madsen Electronics) was used for this procedure. Tonal threshold audiometry consisted of detection of thresholds by means of the air route (frequencies of 250 to 8000 Hz) and the bone route (frequencies of 500 to 4000 Hz), when necessary. The technique used was sound-to-silence, and the threshold was considered to be the lowest intensity at which the child responded 50% of the times to the presence of sound. To classify the degree, we used the mean tonal thresholds per airway at the frequencies of 500 to 2000 Hz and the value proposed by Northern and Downs^10^ for children (mean frequency: normal, up to 15 dB; discrete loss, 16 to 25 dB; mild loss, 26 to 40 dB; moderate loss, 41 to 70 dB; severe loss, 71 to 90 dB; profound loss, more than 91 dB). Since all the children had symmetrical hearing between their ears, as confirmed by the Student t test for paired samples (significance level of 0.05), a single numerical value was used for each frequency evaluated (mean of the values obtained at that frequency for each ear).

### Tympanometry

A Zodiac 901 tympanometer (Madsen Electronics) was used to assess the condition of the middle ear. In the present study, the tympanometric curve was only used for exclusion purposes. Children with type As, Ad, B or C curves on the day of the audiological examination were excluded.

### Staggered Spondaic Word Test (SSW)^11^

We used the Midimate 622 instrument (Madsen Electronics) and band 6 of the volume 2 CD of the Manual of Auditory Processing Assessment,11 placed in a Discman Panasonic SL-SV590W CD player with an adaptor cable for output to an audiometer. This test assesses the auditory skills of auditory memory, figurefundus, binaural integration and auditory closure. For application to the Brazilian population, Katz's SSW test was adapted by Borges, Rejtaman and Schneider under supervision by Katz, in accordance with the basic assumptions of the test, with the same application and analysis. The test was applied at an intensity of 50 dBNS and consisted of presentation of 40 sequences of four words recorded on a CD. All of these words were paroxytone, i.e. the stress was on the penultimate syllable. The presentation of the sequence of words sometimes started with the right ear and at other times with the left ear. Two of these words (second syllable of the second word and first syllable of the third word) were presented simultaneously to the two ears (competitive condition), with partial superimposition. The individual was supposed to repeat the sequence of four words in the order presented. The analysis conducted was both quantitative and qualitative. In the present study, we followed the analysis proposed by the authors of the test adapted to the Portuguese language (analysis of the competitive conditions, auditory effect, order effect, type A effect and inversions).^11^

### Statistical analysis

Descriptive statistical methods were used to characterize the sample. The Kruskal-Wallis test was applied to assess possible differences in audiometric threshold values and in the number of correct responses, under the condition of competitive listening in the SSW test between the three groups (G1, G2 and G3), with the significance level set at 5%. When the null hypothesis was rejected (P-value < 0.05), a post-test was applied in order to determine where the difference was located. This was the Kruskal-Wallis multiple comparisons post-test, which defined the points between which there was a difference among the groups. The test of equality of proportions between two samples was used in order to analyze the variables of effect of order, hearing effect, inversions, type A response and presence/absence of altered auditory processing between the three groups, and to compare some variables relating to the children's medical histories between the groups. Since this test compares two groups at a time (test of equality of proportions between two samples), and since three groups were present, the significance level was reduced based on the formula "α/number of tests", and was set at 1.7% (α = 0.05; number of groups = 3; new significance level = 0.05/3 = 0.017) for this test.

## RESULTS

Table 1 presents the data regarding the children's medical histories, such as prematurity and whether the child was small for gestational age or had a history of repeated otitis or delayed speech development. There was no difference between the groups, thus demonstrating that the groups were similar in these regards.

**Table 1:** Data regarding the children's medical histories

	G1	G2	G3	P-value (G1 x G2)	P-value (G1 x G3)	P-value (G2 x G3)
Prematurity	46%	7%	13%	0.018	0.04	0.5
Small for gestational age (SGA)	20%	13%	13%	0.6	0.06	0.1
History of repeated otitis	26%	40%	46%	0.4	0.2	0.7
Delayed speech development	26%	0%	0%	0.03	0.03	1

Test of equality of proportions between two samples

α = 0.017

G1 = children diagnosed with malnutrition in their first two years of life

G2 = children without history of malnutrition but with learning difficulties

G3 = children without history of malnutrition and without learning difficulties.

Table 2 presents the data on assessment of nutritional status for G1 at the time of diagnosis of malnutrition. The degree of malnutrition, demonstrated by the W/A Z-score, was quite variable (-2.26 to -43.15). The duration of malnutrition ranged from less than one month up to one year and five months of age, thus demonstrating that this group consisted of children with a history of acute or chronic malnutrition. 

**Table 2:** Data on assessments of nutritional status among G1 children at the time of diagnosis of malnutrition

Child	Age (years)	Z-score W/A	Duration of malnutrition (years)
01	1.1	-43.15	1.1
02	0.7	-6.34	0.8
03	0.4	-3.98	1.0
04	0.5	-6.84	1.4
05	0.1	-5.81	1.5
06	0.1	-3.81	0.2
07	0.1	-3.27	> 0.1
08	0.1	-2.38	0.2
09	0.4	-4.6	0.11
10	1.3	-3.54	0.1
11	0.5	-11.21	0.3
12	0.1	-2.96	1.1
13	0.6	-4.54	1.0
14	1.7	-5.81	0.10
15	0.3	-2.26	0.10

G1 = children diagnosed with malnutrition in their first two years of life.

### Figure 1 illustrates the data on the current nutritional status of the three groups. It can be seen that 3 G1 children are again in a malnourished condition. 

**Figure 1:**
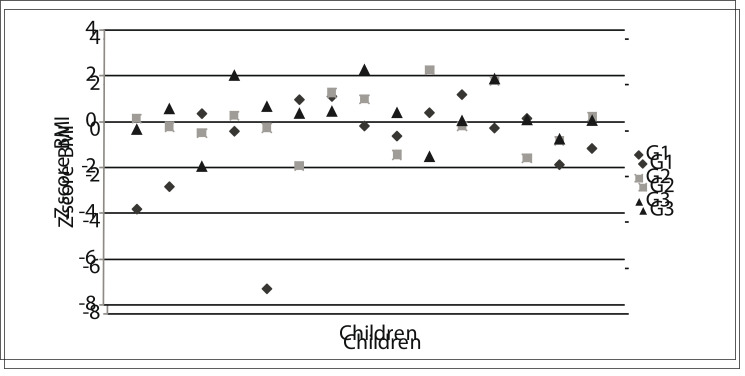
Data on current nutritional status according to the Z-score body mass index (BMI) for each group (G1, G2 and G3).

**Figure 2:**
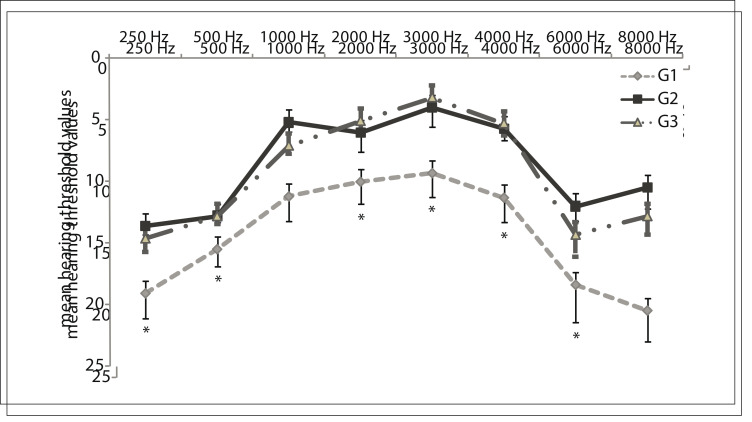
Mean hearing threshold values (Kruskal-Wallis; α = 0.05) for each group (G1, G2 and G3).

Figure 2 shows the mean hearing threshold values. It can be seen that all the children in the three groups had hearing levels within normal patterns. However, when the tonal hearing thresholds were compared between groups, a statistically significant difference was observed between G1 and G2/G3 at six of the other frequencies evaluated. 

### The data obtained through the statistical test are presented in Table 3. Regarding the configuration of the audiometric curve, G1 presented an inverted "U" curve. 

**Table 3:** Data obtained from the statistical test on hearing threshold

	Mean values	P-value	Post-test results
250 Hz	G1 = 19.1	0.03	G1 ≠ G2
	G2 = 13.6		
	G3 = 14.6		
500 Hz	G1 = 15.5	0.3	
	G2 = 12.8		
	G3 = 12.8		
1000 Hz	G1 = 11.2	0.03	G1 ≠ G2
	G2 = 5.2		
	G3 = 7.1		
2000 Hz	G1 = 10	0.1	
	G2 = 6		
	G3 = 5.1		
3000 Hz	G1 = 9.3	0.03	G1 ≠ G3
	G2 = 4		
	G3 = 3.2		
4000 Hz	G1 = 11.3	0.4	G1 ≠ G2
	G2 = 5.7		G1 ≠ G3
	G3 = 5.3		
6000 Hz	G1 = 18.4	0.4	G1 ≠ G2
	G2 = 12		
	G3 = 14.3		
8000 Hz	G1 = 20.5	0.007	G1 ≠ G2
	G2 = 10.5		
	G3 = 12.8		

Kruskal-Wallis test

α = 0.05

G1 = children diagnosed with malnutrition in their first two years of life

G2 = children without history of malnutrition but with learning difficulties

G3 = children without history of malnutrition and without learning difficulties.

**Figure 3:**
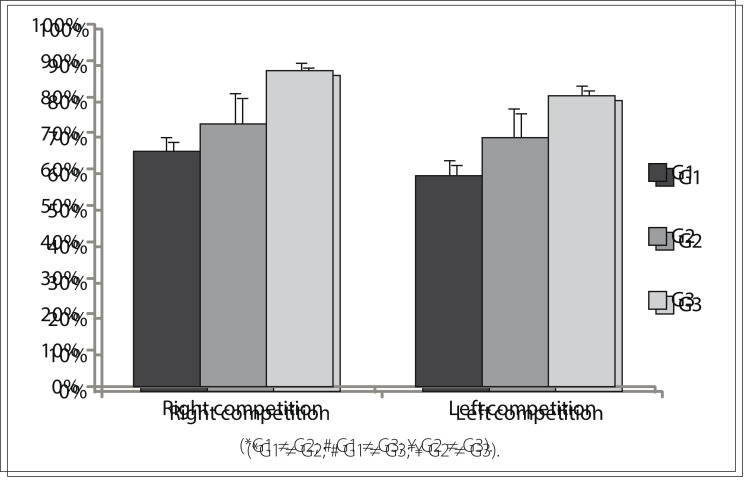
Mean values for each group (G1, G2 and G3) in the Staggered Spondaic Word (SSW) test in the situation of competitive listening.

Figure 3 presents the mean values for each group in the SSW test in situations of competitive listening. It can be seen that G1 and G2 showed similar percentages of correct responses, although both of them responded correctly less than did G3. A statistically significant difference in the test results was observed only between G1 and G3. 

**Figure 4:**
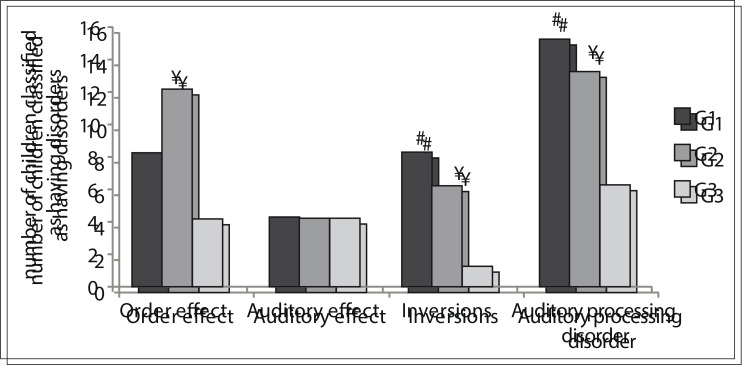
Data from evaluation of the qualitative aspects of Staggered Spondaic Word (SSW) test in each group (G1, G2 and G3).

Figure 4 presents the data on evaluations of the qualitative aspects of the SSW test, which involved the effect order, auditory effect, type A response and number of inversions. This figure also shows the number of children classified as having altered auditory processing. The quantitative and qualitative assessments from the SSW test were taken into consideration in making this classification. It can be seen that the G1 and G2 children had larger numbers of inversions and, at the end of the SSW tests, all the G1 children were classified as presenting altered processing of some auditory skills. A similar proportion of abnormalities was detected in G2. The results from statistical analyses on the groups in the SSW test are listed in Tables 4 and 5.

**Table 4:** Staggered Spondaic Word (SSW) test results for quantitative variables

	Mean score	P-value	Post-hoc analysis
Competitive right	G1 = 64.5%	> 0.001	G1 ≠ G3
	G2 = 71.8%		G2 ≠ G3
	G3 = 87.3%		
Competitive left	G1 = 57.8%	> 0.001	G1 ≠ G3
	G2 = 68.1%		G2 ≠ G3
	G3 = 80.3%		

**Table 5:** Staggered Spondaic Word (SSW) test results for qualitative variables

	Dyad analyzed	P-value
Order effect	G1 x G2	0.1
	G2 x G3	0.003
	G1 x G3	0.1
Auditory effect	G1 x G2	1
	G2 x G3	0.6
	G1 x G3	0.6
Response type A	G1 x G2	1
	G2 x G3	1
	G1 x G3	1
Inversions	G1 x G2	0.4
	G2 x G3	0.03
	G1 x G3	0.005
Auditory processing disorder (SSW classification)	G1 x G2	0.1
	G2 x G3	0.008
	G1 x G3	> 0.001

Comparison test for proportions between two samples

α = 0.017

G1 = children diagnosed with malnutrition in their first two years of life

G2 = children without history of malnutrition but with learning difficulties

G3 = children without history of malnutrition and without learning difficulties.

## DISCUSSION

The results from the present study demonstrate that malnutrition may not be the cause of a large proportion of occurrences of otitis media, but may have a negative effect on the hearing of these children, affecting both the structures of the inner ear and the pathways involved in sound processing.

Although all the G1 children were classified as having audition within normal patterns, this group presented reduced auditory thresholds, compared with the other groups, and an inverted U-shaped audiometric curve. It is important to note that at the time of the evaluation, the children in group G1 had an appropriate nutritional status (except for three who had returned to a state of malnutrition), did not have any damage to the inner ear and presented the same clinical conditions as the other children. This result suggests that malnutrition altered the functioning of the inner ear and caused lasting damage, since 12 of the 15 children in G1 had presented malnutrition only during a single period of life, before reaching two years of age. 

One possible explanation for this finding is that the homeostasis of the inner ear is highly susceptible to various adverse conditions, particularly those of a nutritional nature.^12^ The metabolism of the inner ear is greatly dependent on glucose and oxygen and is intensely active, especially at the level of the vascular stria. However, this organ is practically devoid of energy reserves. Thus, alterations of blood metabolites or lack of proteins, minerals and calories, even in an acute manner, may impair the normal functioning of the inner ear, with negative consequences for the auditory system.^13^ The physiological change caused by malnutrition in the inner ear is also suggested by the type of audiometric configuration detected in G1. The inverted "U" curve is only detected in cases of metabolic alterations.^14^

To describe in detail the changes that occur in the inner ear is a complex task that is often only barely possible because of the great difficulty of access for histological evaluation, especially in humans.^15^ Thus, it is quite difficult to determine in a precise manner what is affected in the inner ear by malnutrition. 

Regarding sound processing, 100% of the G1 children showed alterations in the SSW test. Friederici^1^ reported that the first two years of life are the critical period for development of hearing skills and that development of these skills depends on brain development, which is also at its critical period during this phase. Magalhães et al.^16^ reported that malnourished babies are at risk of developing altered auditory processing, which was confirmed in the present study. These authors observed that malnourished babies showed abnormalities regarding sound location at the age of 12 months. 

Sound location is one of the first auditory processing skills that can be measured, and this skill is affected by maturation, becoming more refined with time until reaching the pattern expected for adult individuals.^17^ Delayed development of this hearing skill suggests that there is a delay in the maturation of the auditory pathways. On this basis, all babies that show this alteration should be monitored in relation to hearing and language, and should undergo a stimulation process when necessary.^18^ Less than 10% of the children included in this study had undergone audiological examination at the time when malnutrition occurred, and therefore it was not possible to determine whether these children already demonstrated abnormalities of auditory skills when they were less than two years old. This result also demonstrates a lack of awareness among health professionals regarding the damage caused to the auditory system by malnutrition, over the short and long terms. Delayed maturation of the auditory pathways, which is one of the causes of auditory processing disorders,^19^ was also reported by Odabas et al.^6^ and Vandana and Tandon^7^ in BAEP performed on malnourished children. 

Lastly, the relationship between the audiometric threshold and auditory processing needs to be discussed. In the present study, it was observed that the children with worse audiometric thresholds were not necessarily the only ones who developed altered auditory processing, given that G2 children with a high incidence of altered auditory processing, along with G1 children, had audiometric thresholds similar to those of G3. This result suggests that the development of auditory skills involves various factors and not simply fluctuating hearing possibly caused by a history of otitis or lower audiometric thresholds. The maturation of auditory processing skills, especially the more complex ones such as those of figure-fundus and binaural fusion, and others, depends on development of other cognitive skills such as working memory.^18^ ^-^ ^22^

During this study, we tried to control for several variables such as socioeconomic level (all the children were from public schools), age, absence of speech stimulation and absence of abnormalities in the inner ear. However, several sources of bias were present, such as the small sample size and the fact that the G1 children had spent time hospitalized during their two first years of life, which may have hampered their initial stimulation and social interaction.

## CONCLUSION

The present findings suggest that malnutrition during the first year of life is a factor involved in a reduction in the audiometric thresholds, even though this does not characterize hearing loss. In the analysis on auditory processing, every child in this group also presented abnormalities of auditory processing abilities.
